# Markers for the population genetics studies of *Triatoma sordida* (Hemiptera: Reduviidae)

**DOI:** 10.1186/s13071-015-0879-1

**Published:** 2015-05-13

**Authors:** Carlota Josefovicz Belisário, Grasielle Caldas D’Ávila Pessoa, Paula Fernandes dos Santos, Letícia Sena Dias, Aline Cristine Luiz Rosa, Liléia Diotaiuti

**Affiliations:** Laboratório de Triatomíneos e Epidemiologia da Doença de Chagas, Centro de Pesquisas René Rachou, Avenida Augusto de Lima, 1715 Barro Preto, CEP 30.190-002 Belo Horizonte, Minas Gerais Brazil

**Keywords:** Triatominae, *Triatoma sordida*, Microsatellites, Population genetic, Chagas disease

## Abstract

**Background:**

*Triatoma sordida*, a vector of *Trypanosoma cruzi*, is native of Brazil, Bolivia, Paraguay, Argentina, and Uruguay, and occurs primarily in peridomiciles. Currently, it is the species most frequently captured by the Chagas Disease Control Program in Brazil. For this reason, population genetic studies attract great interest, as they can provide further information about the dispersal and household invasion processes of this species. In the absence of suitable markers, the objective of this study was to test the cross amplification of microsatellite primers.

**Findings:**

23 primers were tested for microsatellite *loci* already described for other species of the genus *Triatoma* sp. Forty four specimens of *T. sordida* captured in the north of Minas Gerais were used to validate the use of standardized *loci* for population genetic analyses. It was possible to amplify 10 of the 23 loci tested for *T. sordida*.

**Conclusions:**

This is the first study that provides 10 microsatellite markers for population analysis of this triatomine species. Cross-amplification of primers can be used among other phylogenetically related species whose *loci* are already available for study.

## Findings

### Background

*Triatoma sordida* (Stål, 1859) is the triatomine species most frequently captured by Brazil’s Chagas Disease Control Program [1] and in neighboring countries [2]. It occurs in Bolivia, Paraguay, Argentina and Uruguay [2], and Brazilian Cerrado [1]. This vector has broad ecological valence, and so it can live in several ecotopes and use different food sources [3]. It shows high rates of active dispersal [4], and it can also be passively introduced into artificial environments, possibly in firewood piles transported from forests to households, or from one household to another [5], or attached to bird feathers [3]. In an artificial environment, *T. sordida* is often associated with peridomiciles; in addition, it uses birds as its preferred hosts. Thus, this vector has secondary epidemiological importance [1].

Microsatellites are established as a valid technique to study diversity for more than a decade. Because they have highly polymorphic *loci*, they are widely used to investigate the genetic structure of natural populations. These genetic markers possess high reproducibility, multiallelic nature, codominant inheritance, abundance and wide distribution throughout the genome [6].

The use of microsatellites has not been widely applied in triatomines yet, but they are very sensitive markers and yield promising results in the population analyses of this subfamily. Microsatellite markers have been identified and characterized for the following triatomines: *Rhodnius pallescens* [7], *Triatoma dimidiata* [8], *Triatoma infestans* [9, 10], *Rhodnius prolixus* [11, 12], *Triatoma pseudomaculata* [13] and *Triatoma brasiliensis* [14]. Microsatellites described for particular species may sometimes be used to characterize others, e.g., markers for *R. prolixus* that are used for other species of the genus. However, this requires specific research that is certainly associated with the degree of similarity among species [11].

The analysis of gene flow between populations of the wild environment, intradomiciles and peridomiciles can help understand the factors that favor the infestation/reinfestation of the household, and hence provide guidelines for Chagas disease control programs [15, 16]. Considering this fact, the lack of microsatellite primers described for *T. sordida*, and also the great effort required for identifying and characterizing such markers, the objective of this study was to test the amplification of microsatellite *loci* in *T. sordida* using primers described for other triatomine species.

### Methods

Primers were tested for 23 *loci* already described for other species of the genus *Triatoma*: *T. dimidiata* [8], *T. infestans* [10], *T. pseudomaculata* [13] and *T. brasiliensis* [14]. The insects used in the tests were provided from a mixed colony maintained in the insectarium of the Laboratory of Triatomines and Chagas Disease Epidemiology at the René Rachou Research Center. They were originally captured in several northern regions of Minas Gerais. Genomic DNA was extracted from one of the legs of three *T. sordida* adult specimens using the Wizard Genomic DNA Purification Kit (Promega). Quantitation of the DNA was performed in a NanoDrop® ND-1000 spectrophotometer, and the material was kept at −20 °C until processing.

Polymerase chain reactions (PCR) were performed in a final volume of 10 μL containing: 1 unit of Taq DNA Polymerase, Recombinant (Invitrogen), 1x buffer, 1.5 mM or 3 mM of MgCl_2_, 1 mM of dNTP, 5 pmoles of each primer, 2 ng of DNA and ultrapure water. Reactions were performed in a Eppendorf Mastercycles® Gradient thermocycler with the following cycle: initial denaturation at 94 °C for five minutes, 30 cycles at 94 °C for 30 s, temperature gradient dependent on annealing temperature in the primer description (±5 °C) for 30 s and 72 °C for 30 s, followed by final extension at 72 °C for three minutes. The amplified products were visualized in a polyacrylamide gel at 8 % in the mini-gel system (BIO-RAD), stained with 0.2 % silver nitrate.

To determine the size of the *loci*, new PCRs were performed with forward primers tagged with bioluminescent probe. The PCR products were diluted at 1:10 in ultrapure water and genotyped in a MEGABace (Amersham Biosciences) sequencer. The size of the PCR products was estimated in comparison with a standard size marker (ET-400, GE Health Care), and the genotypes were read using the software Fragment Profiler^TM^.

Specimens from the municipality of Coração de Jesus (16°41′15″S, 44°18′45″W), in the north of Minas Gerais, were used to test the use of standardized *loci* for *T. sordida*. These specimens were captured by agents of the Chagas Disease Control Program and not part of the mixed colony used for the selection of microsatellites. Forty four specimens of *T. sordida* were caught in the peridomicile of five neighboring localities: seven in Barriguda, nine in Boa Vista, 10 in Bom Jesus, 10 in Jataí I, and eight in Jataí II.

DNA was extracted from the wing muscle of adult insects [16]. Each muscle was homogenized individually in 100 μL of 1X STE solution (0.01 M NaCl, 0.1 M Tris–HCl and 1 M EDTA), incubated at 90°C for 10 min, and centrifuged at 13,000 rpm for one minute, and the supernatant was recovered. The quantitation and storage of DNA were carried out as described hereinabove.

The samples underwent standard PCR, the annealing temperature and the amount of MgCl_2_ for each primer are described in Table 1. The primers were tagged with bioluminescent probe; genotyping was also performed with standard conditions. For each *locus*, calculations were made of a number of alleles using the software Arlequin 3.1.

**Table 1:** Characteristic of microsatellite loci for T. sordida

*Locus*	Primer sequences (5′- 3′)	T (°C)	MgCl_2_ 50 mM(%)	bp	N
Tb 830	F: GTCAGATGCATGGTGATAC	48	3	119-122(265–292)	4(8)
	R: CATGGAAGATACCTAAACGG				
Tb 8112	F: GAATACGCCTATTCACAG	54	3	78-80(78–96)	3(6)
	R: GGATATGTATTTTAAGGGA				
Tb 8124	F: GCCACTGTGTTCTCATTCC	59.5	3	209-248(209–253)	12(10)
	R: TGGTGTGATGCTCAGAAGG				
TDMS3	F: TCAGATGACGAGGTGGATTG	63	3	137-143(129–146)	3(7)
	R: ACGACCTCAACATCCCTTTC				
TDMS4	F: CAGTTGTTCATCAGGAAGTGAATC	54	6	160(150–186)	1(20)
	R: GCTCAGAAAATATGTTCCCAGT				
Tinfest_ms23	F: CTCTTGCTGGTTGTGCACTG	64	6	156-173(148–177)	10(5)
	R: GTAAACGCCATCCTCACACC				
Tinfest_ms42	F: GACGCTCCAGCTATCGATTC	66	6	205-224(206–246)	9(15)
	R: GGCCAATTGGTTTGGTAG TG				
Tp59	F: ACTTAGGTGGGTATGGA	53	3	121-149(120–128)	9(4)
	R: CAGAGTAGTAGCGTATTGA				
Tp20	F: ACTGACTCCGAGAAAGTG	57	3	125-144(170–206)	7(15)
	R: TTCCTAAATCCAAACCCT				
Tp544	F: TGTTAGAATGAATGCCACTA	55	3	142-213(148–172)	7(8)
	R: GCAATACAATAGAGGACTGA				

*T*, annealing temperature; *bp*, allele size range; *N*, number of alleles. Data in brackets refer to the original description (*Tb*, Harry *et al.* 2009 [14]; *TDMS*, Anderson *et al*. 2002 [8]; *Tinfest_ms*, Marcet *et al*. 2006 [10]; *Tp*, Harry *et al.* 2008 [13])

### Results and discussion

Ten of the 23 pairs of tested primers showed satisfactory amplification for *T. sordida* in specific conditions. The size of the alleles obtained was similar to the original descriptions, except for *loci* Tb830 and Tp20, which were smaller for *T. sordida* (Table 1).

The number of alleles per *locus* ranged from 1 (TDMS4) to 12 (Tb 8124) with a mean of 6.5. Only TDMS4 *locus* showed no heterozygous individuals (Table 1).

Harry *et al.* [11] considered that cross-species amplification should be used with caution because microsatellite null alleles may occur. Their presence should be considered when population analyses are performed, as they may underestimate population diversity [17]. This study could validate the use of the 10 microsatellite markers.

Some of the primers described were tested for cross amplification in other species [10, 11, 13, 14], including *T. sordida* [9]. However, this is the first study that provides microsatellite markers for the population analysis of this triatomine species. Cross-amplification of primers among species was a low-cost, effective strategy which was faster than isolation, identification, and development of primers. Thus, it can be used among other phylogenetically related species whose *loci* are already available for study.

Due to the high sensitivity of the microsatellite markers, this study provides a new tool for the assessment of gene flow between populations of *T. sordida* also in microgeographic scale.
